# Minimum 10 years clinical and radiological outcomes of acetabular revisions of total hip arthroplasties with tricalcium phosphate/hydroxyapatite bone graft substitute

**DOI:** 10.1186/s12891-021-04694-8

**Published:** 2021-09-29

**Authors:** Jacek Gagala

**Affiliations:** Orthopaedic and Traumatology Department, Medical University of Lublin, ul. Jaczewskiego 8, 20-950 Lublin, Poland

**Keywords:** Hip arthroplasty, Revision, Bone graft substitute, Hydroxyapatite, Tricalcium phosphate

## Abstract

**Background:**

Aseptic loosening is the most frequent indication for revision of total hip arthroplasty. Revision arthroplasty of acetabular component is a challenge for every surgeon because they have to simultaneously deal with the reconstruction of bone defects, adequate implant geometry and stable fixation. Allografts are the most frequently used materials in reconstruction of bone loss during revision surgeries. Because of an increasing number of revision hip arthroplasties and poor availability of allografts, we decided to use bone graft substitutes in acetabular revisions.

**Methods:**

Between September 2005 and January 2010, 44 revision arthroplasties in 43 patients were performed with the use of bone graft substitutes for acetabular defect reconstruction in revision of total hip arthroplasty. Acetabular bone defects were classified according to Paprosky. Seventeen hips were classified as IIA, 3 hips IIB, 3 hips IIC, 10 hips IIIA and 11 hips IIIB. Acetabular bone defects were reconstructed with tricalcium phosphate/hydroxyapatite bone graft substitute - BoneSave. Clinical and radiological examination was performed after 3 months, 1 year and then annually. Harris hip score was used for clinical evaluation. Survival analysis was performed with Kaplan-Meier method with aseptic loosening as the definition of endpoint.

**Results:**

The average follow-up period is 12 (range from 10 to 15) years. During the follow-up, three patients died after 24 months because of causes not related to surgery. None of the patients was lost to follow-up. The evaluation of clinical results revealed an increase in pre-operative HHS from average 38.3 (range 25 to 55) points to average 86.3 (range 45 to 95) points at the most recent follow-up. Radiographic evaluation showed the migration of one revision cage 12 months after surgery. Revision arthroplasty performed after 14 months revealed the partial incorporation of bone graft substitute. There were not any cases of loosening of revision acetabular cup at the most recent follow up examination in the remaining 39 patients. Bone graft substitute was not absorbed in all of these patients. The survival after 10 years amounted to 97.56%.

**Conclusion:**

Bone graft substitute Bone Save may be suitable for acetabular revision surgery, however preoperative bone defect is critical for success and determining of a surgical technique, so this is multifactorial in this challenge surgery.

**Supplementary Information:**

The online version contains supplementary material available at 10.1186/s12891-021-04694-8.

## Background

Aseptic loosening is the most frequent indication for revision of total hip arthroplasty [1]. Other indications include: periprosthetic osteolysis, wear, instability, infection, breakage of bearing surfaces and periprosthetic fracture [1]. Revision arthroplasty of acetabular component is a challenge for every surgeon because they have to simultaneously deal with the reconstruction of bone defects, adequate implant geometry and stable fixation [2]. Depending on the extent of the missing bone, the variety of possible implants range from primary cemented [3] or uncemented cups [2, 4, 5], oblong cups [6], antiprotrusio cages [3, 7], highly porous implants with augments and cup-cage constructs [2, 5]. Bone autograft is the gold standard in the treatment of various defects because of osteoconductive and osteogenic properties, but its possible application in the treatment of large lesions in revision hip surgery is limited due to availability. Other problems connected with autografts are: donor site morbidity, increase in operative time, possible vascular and neurological complications, increase in blood loss and postoperative pain. Allografts are the most frequent materials used in reconstruction of bone loss during revision surgeries [2–4, 6, 8]. Although the application of allograft has very good survival rates, there is always a risk of transmission of viral or bacterial infection and antigenicity. The process of preparation of allograft minimizes the possible infection or host’s immune response, but decreases mechanical properties and increases the risk of implant migration and loosening [3]. BoneSave is biphasic ceramic porous bone graft substitute, which consists of 80% of tricalcium phosphate (TCP) and 20% hydroxyapatite (HA) [9, 10]. This composition is very close to the mineral phase of bone. BoneSave (Stryker, Mahwah, NJ, USA) is indicated as a graft extender that should be mixed with auto or allograft in the proportion of 1:1, and has been designed for revision impaction grafting both for the acatabulum and femur. Because of an increasing number of revision hip arthroplasties and poor availability of allografts, we decided to use BoneSave alone in acetabular revisions. The purpose of this study is a retrospective clinical and radiological evaluation of bone graft substitute BoneSave in acetabular reconstructions during revision of total hip arthroplasty.

## Methods

One hundred thirty-seven revision operations were performed between January 2005 and January 2010 due to aseptic loosening of acetabular components of total hip replacements. In 44 (32.1%) operations bone graft substitute BoneSave was used for acetabular bone stock reconstruction. The indication for BoneSave was large contained defect of acetabulum. The same indication was for the reconstruction with morsellized allografts. Between January 2005 and March 2006 allografts were used in revision operations of 24 hips (17.5%). Since April 2006 allografts were not available and completely replaced by BoneSave. The study group consisted of 18 women (19 hips) and 25 men with the average age of 68.58 (range 40 to 83) years at the time of operation (Table 1). Mean survival of primary implant was 11.8 (range 3 to 25) years. In 41 patients (42 hips) it was the first revision surgery, in two patients - the second one. The indication of revision surgery was aseptic loosening of 33 cemented and 10 uncemented cups. One patient was operated on because of acetabular devastation after Austin-Moore hemiarthroplasty. Acetabular revision alone was performed in 21 patients (22 hips), while revision of both components in the remaining 22 patients. In four patients, open reduction and internal fixation of femoral periprosthetic fracture was performed during the same procedure with the use of Dall/Miles trochanteric plates and cables (Stryker, Mahwah, NJ, USA). Acetabular bone defects were classified according to Paprosky [11]. Seventeen hips were classified as IIA, 3 hips IIB, 3 hips IIC, 10 hips IIIA and 11 hips IIIB. Three different approaches were used during revision surgeries: postero-lateral in 28 patients (29 hips), trochanteric slide osteotomy with the use of anterior hip joint exposure in 12 patients and extended femoral osteotomy with anterior hip joint exposure in the remaining 3 patients. After primary cup had been removed, and scar and granuloma tissue debrided, the extent of acetabular defect was evaluated. Average 1.3 (range 1 to 3) 40 g package of 4–6 mm granule size of BoneSave was used for acetabular reconstruction. After BoneSave had been poured out of the packing to the bowl the spatula was used to mix it with blood harvested from patient’s operative field. Bone graft substitute was compressed to acetabular defect with reverse reaming technique. Care was taken to achieve fill of the osteolitic lesion with adequate volume of bone graft substitute, its firm compression and stable fixation of definitive implant. The type of revision socket used depended on pre-operative Paprosky stage as well as on intra-operative bone defects examination. Cemented Exeter cup (Stryker, Mahwah NJ, USA) was used in 4 patients. Uncemented sockets were implanted in 20 patients (21 hips): Trident (Stryker, Mahwah, NJ, US) cup in 17, Regenerex (Biomet, Warsaw, IN, USA) in 1, Screwcup (BBraun Aesculap, Tuttlingen, Germany) in two patients (3 hips). Antiprotrusio cages were implanted in patients with Paprosky III stage: Recon Cage (BBraun Aesculap, Tuttlingen, Germany) in 3 patients, Burch-Schneider cage (Zimmer, Warsaw, IN, USA) in 16 patients. Partial weight bearing with two crutches started on the third postoperative day and advanced to full weight bearing after 12 weeks. Clinical and radiological examination was performed after the period of 3 months, 1 year and then annually. Harris hip score (HHS) was used for clinical evaluation [12]. X-ray examination was performed with the use of AP view of both hips. Immediate postoperative and most recent radiographs were compared to evaluate revision cup stability, the evidence of radiolucence lines and incorporation of bone graft substitute. A line connecting tear drops was drawn on plain radiographs. The vertical distance from the center of endoprosthetic head to inter-teardrop line was measured for superior migration. The horizontal distance from the center of the prosthetic head to Köhlers line was measured for medial migration. Medial or superior migration greater than 5 mm was defined as a sign of revision cup instability. Radiolucency around revision cups was quantified as > 2 mm in three zones according to DeLee and Charnley [13]. Brooker classification was used for heterotopic ossification staging [14]. The differences in pre-operative and post-operative HHS was evaluated with Wilcoxon paired samples test. Kaplan-Meier survival analysis was carried out with revision surgery as definition of failure. All statistical analysis was performed with Statistica 1.3 (Statsoft Inc.).

**Table 1:** Patient demographic and Paprosky classification

Mean age (range, years)	
Male	69 (44–83)
Female	68 (40–83)
Gender (no. of patients, hips)	
Male	25
Female	18 (19)
Paprosky classification	
IIA	17
IIB	3
IIC	3
III A	10
IIIB	11

The Ethics Committee at Medical University of Lublin (KE-0254/129/2020) approved the study. All methods were carried out in accordance with relevant guidelines and regulations. Informed consent was obtained from all the patients.

## Results

The average follow-up is 12 (range 10 to 15) years. During the follow-up, three patients died after 24 months because of causes not related to surgery. None of the patients was lost to follow-up.

The evaluation of clinical results revealed an increase in pre-operative HHS from average 38.3 (range 25 to 55) points to average 86.3 (range 45 to 95) points at the most recent follow-up. The differences of pre-operative and post-operative HHS were statistically significant (*P* < 0,001). There were not any cases of deep infection of operated hip, or any signs of immunization.

**Fig. 1:**
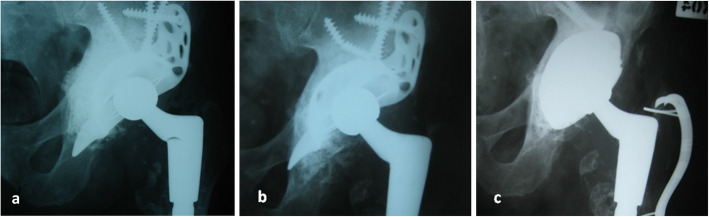
**a** Postoperative radiograph after reconstruction of acetabular defects with tricalcium phosphate/hydroxyapatite bone graft substitute and antiprotrusio cage in 77 year-old patient. A minor medial displacement of BoneSave particles is presented, caused by intraoperative crack during its compression. **b** Cage migration after 12 months, graft is compressed and partially incorporated. **c** After re-revision with oblong cup

**Fig. 2:**
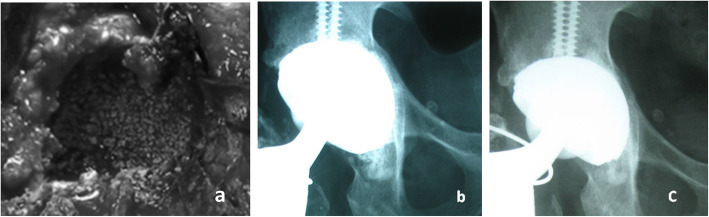
**a** Intraoperative view of acetabular reconstruction with bone graft substitute in 44 year-old patient. **b** Postoperative radiograph presents well-fixed socket with impacted bone graft substitute. **c** After 10 years acetabular cup is still well-fixed with graft bonded with host bone

**Fig. 3:**
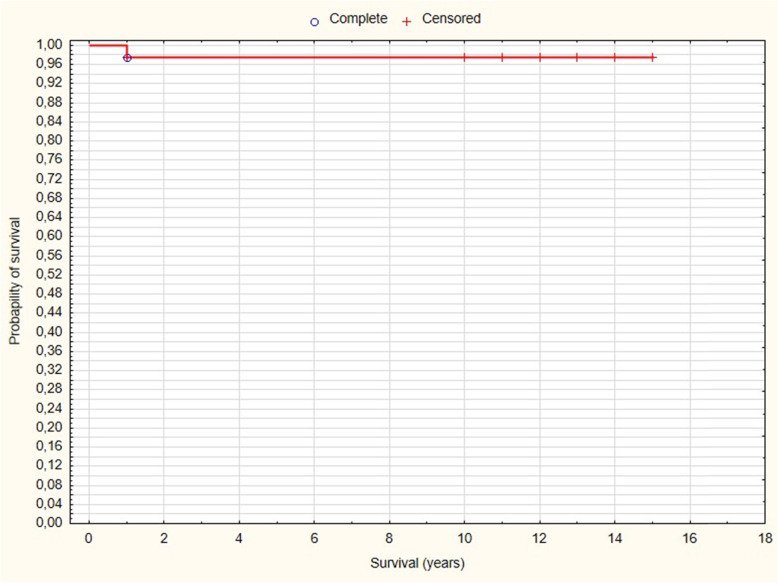
Kaplan-Meier survival plot. 97.56% survival was noted after 10 years

Radiographic evaluation showed the migration of one Burch-Schneider cage 12 months after surgery (Fig. 1). Revision arthroplasty was performed after the period of 14 months. Partial incorporation of bone substitute was observed during this operation. The implantation of Procotyl®E (Wright Medical, Arlington, TN, USA) revision cup proceeded in this patient without any complications. Bone graft substitute was observed not to be absorbed in the remaining 39 patients (Fig. 2). There were not any cases of loosening of revision acetabular cup at the most recent follow up examination. None of the patients complained about any allergic reaction related with revision surgery. The survival after 10 years performed with Kaplan-Meier analysis was 97.56% with aseptic loosening as definition of endpoint (Fig. 3). Eleven cases of heterotopic ossification were noted: Brooker I in 8 patients, Brooker II in 2 and Brooker III in the remaining one patient. One patient had early postoperative dislocation, which was treated with closed reduction, and immobilized in hip cast for 8 weeks. No further recurrent dislocation occurred in this patient. Two other patients had revision surgeries not related to the revised cup - one because of periprosthetic femoral shaft fracture 48 months after the surgery. Open reduction and internal fixation with plate and cables was performed. The fracture healed within 8 months. The second patient was operated on due to the breakage of metal cable fixing healed femoral osteotomy. The cable was removed and patient has a good clinical result.

## Discussion

Bone graft substitute should promote bone healing with a new bone formation, without the risk of transmission of infection or immunological host response [9, 10]. Bone graft substitute should also be reproducible and cost effective in comparison to allograft. It was reported that bone graft substitutes had been successfully used in the treatment of defects due to bone cysts, avascular necrosis, scoliosis and trauma [15–19]. Bone graft substitute used for revision surgery should provide an adequate stability and support for the implant [9, 10]. Bolder et al. reported on initial stability of cemented cups in acetabular defects reconstructed with impacted morsellized bone grafts or TCP/HA granules in vitro [20]. Reconstructions with the TCP/HA granules demonstrated the highest stability of the cups. TCP/HA particles mixed with bone grafts showed better stability than bone grafts alone. Similar results of mechanical engineering testing as well as animal model were presented of femoral reconstructions with the use of TCP/HA bone graft substitutes [11, 21]. The papers presenting outcomes of bone grafts substitutes used in bone stock reconstruction during revision hip arthroplasty differ in relation to the type of material used and include as follows: xenograft [22, 23], glass ionomer ceramics [9], corraline derived hydroxyapatite [24], calcium sulphate [18, 25], HA [7, 26–28], TCP [7, 29] and TCP/HA [10, 30–34]. There is also a lack of reports presenting a large population and randomized trials. These substances may be used alone or in conjunction with auto or allograft. Xenografts were reported to incorporate worse than allografts and had more cases of infection [22]. Glass ionomer neither resorbs nor has the potential for replacement with host bone [9]. Wasielewski et al. reported the use of corraline derived hydroxyapatite alone or in combination of either autograft or allograft in 17 acetabular reconstructions [24]. After a mean follow-up of 49 months the authors noted bone incorporation in all reconstructed hips. One loosening of socket occurred in the patient treated due to severe osteolysis. Calcium sulphate resorbs quickly, and it was reported that the application of calcium sulphate cement demonstrates good results in filling osteolytic defects around stable uncemented cups [25]. Stravinskas et al. presented early results of application of Cerament^R^G injectable bone substitute consisting of hydroxyapatite, calcium sulphate and gentamycin in treatment of revision hip arthroplasty in 10 patients [18]. Cerament G was applied to reconstruct bone defects in proximal femur in conjunction with uncemented modular stems. Bone graft healing was observed after 3 months in all patients.

HA, TCP or the combination of HA/TCP is the most frequent osteoconductive bone grafts substitute used in revision hip arthroplasty [9]. Synthetic HA is a non-resorbing material and HA particles bind together and to host bone by physicochemical reaction [26]. TCP is a resorbing material, which is stronger, stiffer and tougher than HA [9, 35]. Combining these two materials forms Biphasic TCP. Desorption rate of biphasic TCP is dependent on HA/TPC ratio [9, 10]. The composition of 80% of TPC and 20% of HA is very close to the mineral phase of bone. Biphasic granules of TCP/HA have pores whose size has been revealed to be optimal for osteoconduction. TCP/HA granules are manufactured in particulate form with harder consistency than morsellized allograft to withstand impaction grafting forces and enhance the initial stability of the implant [10, 20, 21, 36]. TCP/HA particles are slowly replaced by host bone [34]. The migration of bone graft substitute granules between endoprosthetic bearing surfaces may cause increased wear and osteolysis.

Oonishi and colleagues used HA particles exclusively in revision arthroplasties of 40 massive bone defects [26]. After the period of from 4 to 10 years they reported direct bonding of HA to host bone. Three cups migrated and had been loosened. McNamara et al. used 1:1 mixture of frozen irradiated allografts with HA granules in 37 revision and 13 complex primary acetabular reconstructions [27]. After a mean follow- up of 60 months’ period the authors reported 100% survival. The initial migration was revealed in two cups before stabilizing. Aulakh et al. compared results of 42 acetabular revisions with allograft with 23 reconstructions performed with a combination of HA granules mixed with allograft [28]. Both groups of patients did not differ in relation to clinical results, complications and survival at 13 years. The authors concluded that the use of bone graft substitute with allograft is comparable to allograft alone.

Nich and Sedel reported the outcomes of 21 femoral revisions with the application of TCP granules exclusively, with allograft or autograft in the treatment of femoral defects [29]. After an average follow-up of 36 months the authors reported the absence of radiological osteolysis in 17 hips, which suggested direct bonding between TCP granules and host bone.

Schwartz and Bordei used TCP/HA particles in 32 acetabular revisions [30]. Bone graft substitute granules consisted of 35% TCP and 65% HA. Uncemented hemispherical cups were used for cavitary defects, while antiprotrusio cages for large segmental defects. After average of 5.5 years the authors noted no specific complications, and the incidence of dislocations and infections was similar to other series. Haenle et al. used HA/TCP bone graft substitute (80% TCP, 20% HA) with conjunction of collagen-hydroxyapatite fleece for acetabular defect reconstruction during 22 revision hip arthroplasties [31]. No bone graft was used. Uncemented cranial socket was implanted in all of the cases. After average of 20.5 months there were not any signs of implant loosening and increase in clinical scores. Blom et al. reported the outcomes of acetabular revision with impacted bone grafts in conjunction with BoneSave in 43 patients [10]. Nine cemented and 34 uncemented cups were used. After average of 24 months there were no re-revisions, and no implant migrations. The patients had very good clinical results and were very satisfied with the procedure. In 2013 Whitehouse et al. reviewed the same cohort of patients [32]. The survival of grafted acetabulum and acetabular component was 94% at the period of 7 years. One patient had been revised for aseptic loosening and 1 for deep infection. Graft material became incorporated in all 3 zones of the acetabulum in 23 out of 24 cases that had complete radiographic follow-up. In another paper, Whitehouse reported on the use of BoneSave alone for reconstruction of contained defects of acetabulum [33]. A cohort of 43 patients was reviewed with a mean follow-up of 4 years. The viability of the acetabular component was 98% at 7 years. There was one revision for infection and one radiographic failure. The graft substitute had incorporated in all 3 zones of the acetabulum in 33 of 37 cases with complete radiographic follow-up. The authors concluded that BoneSave alone is reliable material for impaction grafting of contained defects in the acetabulum at revision surgery.

Patients from present study have good clinical outcomes as compared to the previously cited reports. No cases of infection or antigenicity similar to present study were reported either with TCP/HA alone or in conjunction with allograft. There was one case of early re-revision in our group of patients. The cause of failure presented in this study is due to a technical error in implantation of antiprotrusio cage and should not be attributed to bone graft substitute. The proportion of postoperative complications in present report is similar to other papers reviewing the outcomes of bone graft substitutes. The limitation of present study is that it is retrospective, not randomized and includes a small number of patients operated with different implants.

## Conclusion

Bone graft substitute Bone Save may be suitable for acetabular revision surgery, however preoperative bone defect is critical for success and determining of a surgical technique, so this is multifactorial in this challenge surgery.

## Supplementary Information

**Additional file 1.**

## Data Availability

All data generated or analyzed during this study are included in this published article and its supplementary information files.

## References

[1] Ulrich SD, Seyler TM, Bennett D, Delanois RE, Saleh KJ, Thongtrangan I, Kuskowski M, Cheng EY, Sharkey PF, Parvizi J, Stiehl JB, Mont MA (2008). Total hip arthroplasties: what are the reasons for revision?. Int Orthop.

[2] Pulido L, Rachala SR, Cabanela ME (2011). Cementless acetabular revision: past, present, and future. Int Orthop.

[3] Kowalczewski JB, Rutkowska-Sak L, Marczak D, Słowińska I, Słowiński R, Skibiński M (2013). Bone graft incorporation after revision hip arthroplasty in patients with rheumatoid arthritis, seventy eight revisions using bone allografts with or without metal reinforcements. Int Orthop.

[4] Pereira GCT, Kubiak EN, Levine EN, Chen FS, Di Cesare PE (2007). Cavitary acetabular defects treated with morselized cancellous bone graft and cementless cups. Int Orthop.

[5] Theil C, Schmidt-Braekling T, Gosheger G, Moellenbeck B, Schwarze J, Dieckmann R (2019). A single center study of 41 cases on the use of porous tantalum metal implants in acetabular revision surgery. BMC Musculoskelet Disord.

[6] Civinini R, Capone A, Carulli C, Villano M, Gusso MI (2008). Acetabular revision using a cementless oblong cup: five to ten year results. Int Orthop.

[7] Hayashi S, Nishiyama, Hashimoto S, Matsumoto T, Takayama K, Ishida K, Nishida K, Kuroda R (2017). Risk factors for failure of revision total hip arthroplasty using a Kerboull-type acetabular reinforcement device. BMC Musculoscelet Disord.

[8] Xiao Q, Wang H, Zhou K, Wang D, Ling T, Pei F, Zhou Z (2019). The mid-long term results of reconstructional cage and morselized allografts combined application for the Paprosky type III acetabular bone defects in revision hip arthroplasty. BMC Muskuloskelet Disord.

[9] Beswick A, Blom AW (2011). Bone graft substitutes in hip revision surgery: a comprehensive overview. Injury Int Care Injured.

[10] Blom AW, Wylde V, Livesey C, Whitehouse MR, Eastaugh S, Bannister GC, Learmonth ID (2009). Impaction bone grafting of the acetabulum at hip revisions using a mix of bone chips and a biphasic porous ceramic bone graft substitute. Acta Orthop.

[11] Paprosky WG, Perona PG, Lawrence JM (1994). Acetabular defect classification and surgical re construction in revision arthroplasty: a 6-year follow-up evaluation. J Arthroplast.

[12] Harris WH (1969). Traumatic arthritis of the hip after dislocation and acetabular fractures: treatment by mold arthroplasty. An endresult study using a new method of result evaluation. J Bone Joint Surg Am.

[13] DeLee JG, Charnley J (1976). Radiological demarcation of cemented sockets in total hip replacement. Clin Orthop Relat Res.

[14] Brooker AF, Bowerman JW, Robinson RA, Riley LH (1973). Ectopic ossification following total hip replacement: incidence and a method of classification. J Bone Joint Surg Am.

[15] Kaczmarczyk J, Sowinski P, Goch M, Katulska K (2015). Complete twelve month bone remodeling with a bi-phasic injectable bone substitute in benign bone tumors: a prospective pilot study. BMC Musculoscelet Disord.

[16] Landgraeber S, Warwas S, Claβen, Jäger M (2017). Modifications to advanced core decompression for treatment of avascular necrosis of the femoral head. BMC Musculoskelet Disord.

[17] Fu T-S, Wang I-C, Lu M-L, Hsieh M-K, Chen L-H, Chen W-J (2016). The fusion rate of demineralized bone matrix compared with autogenous iliac bone graft for long multi-segment posterolateral spinal fusion. BMC Musculoskelet Disord.

[18] Stravinskas M, Tarasevicius S, Laukaitis S, Nilsson M, Raina B, Lidgren L (2018). A ceramic bone substitute containing gentamicin gives good outcome in trochanteric hip fractures treated with dynamic hip screw and in revision of total hip arthroplasty: a case series. BMC Musculoskelet Disord.

[19] Iundusi R, Gasbarra E, D’Arienzo M, Piccioli A, Tarantino U (2015). Augmentation of tibial plateau fractures with an injectable bone substitute Cerament. Three year follow-up from a prospective study. BMC Musculoskelet Disord.

[20] Bolder SBT, Verdonschot N, Schreurs BW, Buma P (2002). Acetabular defect reconstruction with impacted morsellized bone grafts or TCP/HA particles. A study on the mechanical stability of cemented cups in an artificial acetabulum model. Biomaterials.

[21] Munro NA, Downing MR, Meakin JR, Lee AJ, Ashcroft GP (2006). A hydroxyapatite graft substitute reduces subsidence in a femoral impaction grafting model. Clin Orthop Relat Res.

[22] Charalambides C, Beer M, Cobb AG (2005). Poor results after augmenting autograft with xenograft (Surgibone) in hip revision surgery: a report of 27 cases. Acta Orthop.

[23] Levai JP, Boisgard S (1996). Acetabular reconstruction in total hip revision using a bone graft substitute. Early clinical and radiographic results. Clin Orthop Relat Res.

[24] Wasielewski RC, Sheridan KC, Lubbers MA (2008). Coralline hydroxyapatite in complex acetabular reconstruction. Orthopaedics.

[25] Engh CA, Egawa H, Beykirch SE, Hooper RH, Engh CA (2007). The quality of osteolysis grafting with cementless acetabular retention. Clin Orthop Relat Res.

[26] Oonishi H, Iwaki Y, Kin N, Kushitani S, Murata N, Wakitani S, Imoto K (1997). Hydroxyapatite in revision of total hip replacements with massive acetabular defects: 4- to 10- year clinical results. J Bone Joint Surg Br.

[27] McNamara I, Deshpande S, Porteous M (2010). Impaction grafting of the acetabulum with a mixture of frozen, ground irradiated bone graft and porous synthetic bone substitute. J Bone Joint Surg Br.

[28] Aulakh T, Jayasekera N, Kuiper J, Richardson J (2009). Long-term clinical outcomes following the use of synthetic hydroxyapatite and bone graft in impaction in revision hip arthroplasty. Biomaterials.

[29] Nich C, Sedel L (2006). Bone substitution in revision hip replacement. Int Orthop.

[30] Schwartz C, Bordei R (2005). Biphasic phosphor-calcium ceramics used as bone substitutes are efficient in the management of severe acetabular bone loss in revision total hip arthroplasties. Eur J Orthop Surg Traumatol.

[31] Haenle M, Schlüter S, Ellenrieder M, Mittelmeier W, Bader R (2013). Treatment of acetabular defects during revision total hip arthroplasty- preliminary clinical and radiological outcome using bone substitute material. Hip Int.

[32] Whitehouse MR, Dacpmbe PJ, Webb JCJ, Blom AW (2013). Impaction grafting of the acetabulum with ceramic bone graft substitute mixed with femoral head allograft: high survivorship in 43 patients with a median follow-up of 7 years. A follow-up report. Acta Ortop.

[33] Whitehouse MR, Dacombe PJ, Webb JCJ, Blom AW (2013). Impaction grafting of the acetabulum with ceramic bone graft substitute. High survivorship with a mean follow-up period of 4 years. Acta Orthop.

[34] Fujishiro T, Nishikawa T, Niikura T, Takikawa S, Saegusa Y, Kurosaka M, Bauer TW (2008). Histologic analysis of allograft mixed with hydroxyapatite-tricalcium phosphate used in revision femoral impaction bone grafting. Orthopaedics.

[35] Nishii T, Sugano N, Miki H, Koyama T, Yoshikawa H (2006). Multidetector-CT evaluation of bone substitutes remodeling after revision hip surgery. Clin Orthop Relat Res.

[36] Coathup M, Smith N, Kingsley C, Buckland T, Dattani R, Ascroft P, Blunn G (2008). Impaction grafting with a bone-graft substitute in a sheep model of revision hip replacement. J Bone Joint Surg Br.

