# Supplementary material for: Minimum 10 years clinical and radiological outcomes of acetabular revisions of total hip arthroplasties with tricalcium phosphate/hydroxyapatite bone graft substitute
Source: BMC Musculoskelet Disord. 2021 Sep 29;22:835. doi: 10.1186/s12891-021-04694-8 (PMC8480101; doi:10.1186/s12891-021-04694-8)
Supplement: Supplementary file 1 — Additional file 1. [file 12891_2021_4694_MOESM1_ESM.docx]

Clinical and radiological outcomes of acetabular revisions of total hip arthroplasties with tricalcium phosphate/hydroxyapatite bone graft substitute. Forty patients with minimum follow-up of 10 years.

Jacek Gagala

Orthopaedic and Traumatology Department, Medical University of Lublin, Lublin, Poland

Corresponding Author: Jacek Gagala MD, Orthopaedic and Traumatology Department, Medical University of Lublin, ul. Jaczewskiego 8, 20-950 Lublin, Poland; [jacekgagala@gmail.com](mailto:jacekgagala@gmail.com)

| Patient | gender | Case hist. No. | Year of revision | Component to be revised | Age | HHS before op | Paprosky classification | years after previous surgery | Previous implant | No of previous operations | Revision implant | Complications | survival | Follow up / years |
| --- | --- | --- | --- | --- | --- | --- | --- | --- | --- | --- | --- | --- | --- | --- |
| TE | F | 500105 | 2005 | CUP | 55 | 42 | IIIB | 12 | THREADED | 1 | AESCULAP | Fracture femur | 0 | 15 |
| PW | F | 280216 | 2005 | CUP | 77 | 40 | IIIB | 12 | CEMENT | 1 | AESCULAP | N | 0 | 15 |
| SL | M | 520101 | 2005 | CUP | 53 | 52 | IIIB | 8 | THREADED | 1 | AESCULAP | N | 0 | 15 |
| IJ | M | 241005 | 2005 | BOTH | 81 | 45 | IIA | 15 | CEMENT | 1 | CEMENT | N | DEATH |  |
| LZ | M | 530930 | 2006 | BOTH | 53 | 40 | IIB | 16 | CEMENT | 3 | CEMENT | N | 0 | 14 |
| KJ | M | 570422 | 2006 | BOTH/ FRACTURE | 59 | 50 | IIA | 9 | CEMENT | 1 | TRIDENT | N | 0 | 14 |
| KW | M | 301210 | 2007 | BOTH | 77 | 40 | IIIB | 14 | CEMENT | 2 | BS | Cup loosening | 1 | 1 |
| WK | M | 630222 | 2007 | BOTH | 44 | 45 | IIA | 7 | Press fit | 1 | Press fit | N | 0 | 13 |
| DK | F | 371227 | 2007 | BOTH | 70 | 32 | IIIB | 6 | CEMENT | 2 | BS | N | 0 | 13 |
| OS | F | 301102 | 2007 | BOTH | 77 | 41 | IIA | 20 | CEMENT | 2 | Press fit | N | 0 | 13 |
| SE | F | 460409 | 2007 | BOTH | 62 | 38 | IIIB | 8 | CEMENT | 1 | BS | N | 0 | 13 |
| KA | M | 521112 | 2007 | BOTH/ FRACTURE | 55 | 55 | IIA | 10 | CEMENT | 2 | Press fit | N | 0 | 13 |
| UK | M | 400324 | 2007 | CUP | 67 | 40 | IIIA | 9 | CEMENT | 1 | Press fit | Fracture femur | 0 | 13 |
| LM | M | 510105 | 2007 | CUP | 56 | 40 | IIIB | 9 | THREADED | 2 | Press fit | N | 0 | 13 |
| WJ | M | 401207 | 2007 | CUP | 67 | 40 | IIA | 12 | press fit | 1 | Press fit | N | 0 | 13 |
| DJ | F | 310625 | 2007 | CUP | 77 | 35 | IIIB | 8 | CEMENT | 3 | BS | N | 0 | 13 |
| KT | M | 251119 | 2007 | CUP | 72 | 40 | IIIB | 14 | CEMENT | 2 | BS | N | 0 | 13 |
| WA | M | 350202 | 2007 | BOTH/ FRACTURE | 72 | 40 | IIIB | 14 | CEMENT | 3 | BS | N | 0 | 13 |
| GZ | F | 540627 | 2008 | BOTH | 54 | 50 | IIA | 21 | THREADED | 1 | CEMENT | N | 0 | 12 |
| HH | F | 340327 | 2008 | BOTH | 74 | 27 | IIIA | 22 | CEMENT | 3 | BS | N | 0 | 12 |
| MH | F | 330826 | 2008 | BOTH | 75 | 25 | IIIB | 8 | CEMENT | 1,5 | BS | N | 0 | 12 |
| TR | F | 570620 | 2008 | CUP | 51 | 40 | IIB | 14 | press fit | 2 | CEMENT | N | 0 | 12 |
| KJ | M | 420414 | 2008 | CUP | 66 | 37 | IIB | 7 | CEMENT | 1 | Press fit | N | 0 | 12 |
| PS | M | 290413 | 2008 | CUP | 79 | 40 | IIA | 14 | CEMENT | 1 | Press fit | N | DEATH |  |
| KH | F | 320304 | 2008 | CUP | 76 | 45 | IIA | 5 | CEMENT | 1 | Press fit | N | 0 | 12 |
| KH | F | 451108 | 2008 | BOTH | 63 | 35 | IIIA | 11 | CEMENT | 1 | BS | N | 0 | 12 |
| OJ | F | 680519 | 2008 | CUP | 40 | 40 | IIIA | 14 | THREADED | 3 | THREADED | N | 0 | 12 |
| OJ |  | 680519 | 2009 | CUP |  | 40 | IIIA | 15 | THREADED | 2 | THREADED | N | 0 | 12 |
| JJ | M | 390706 | 2008 | BOTH | 69 | 39 | IIA | 9 | CEMENT | 1 | Press fit | N | 0 | 12 |
| KH | F | 460601 | 2008 | CUP | 62 | 38 | IIIA | 10 | CEMENT | 1,5 | BS | N | 0 | 12 |
| PA | M | 301217 | 2008 | CUP | 78 | 40 | IIA | 12 | CEMENT | 1 | Press fit | N | 0 | 12 |
| BT | M | 251018 | 2008 | CUP | 83 | 35 | IIC | 11 | CEMENT | 1 | THREADED | N | 0 | 12 |
| WS | M | 290328 | 2009 | CUP | 80 | 42 | IIA | 15 | CEMENT | 1 | Press fit | N | DEATH |  |
| CE | M | 300828 | 2009 | CUP | 79 | 32 | IIC | 3 | CEMENT | 1 | CUPCAGE | N | 0 | 11 |
| OR | M | 441223 | 2009 | BOTH | 64 | 42 | IIA | 7 | CEMENT | 1 | Press fit | N | 0 | 11 |
| SS | M | 361001 | 2009 | CUP | 73 | 35 | IIA | 11 | CEMENT | 1 | Press fit | N | 0 | 11 |
| SK | M | 360622 | 2009 | CUP | 73 | 42 | IIA | 15 | Press fit | 1 | Press fit | N | 0 | 11 |
| PS | M | 370601 | 2009 | BOTH/ FRACTURE | 72 | 40 | IIA | 22 | CEMENT | 2 | BS | N | 0 | 11 |
| PJ | M | 260408 | 2009 | CUP | 83 | 40 | IIIA | 16 | CEMENT | 1 | BS | N | 0 | 11 |
| GA | F | 410101 | 2009 | BOTH | 68 | 30 | IIIA | 25 | CEMENT | 3 | BS | N | 0 | 11 |
| SM | F | 260223 | 2009 | BOTH | 83 | 32 | IIA | 14 | CEMENT | 1 | Press fit | N | 0 | 11 |
| SJ | F | 300621 | 2009 | CUP | 79 | 30 | IIIA | 3 | CEMENT | 1 | BS | N | 0 | 11 |
| SF | F | 340102 | 2009 | OBA | 75 | 39 | IIIA | 9 | CEMENT | 2 | BS | N | 0 | 11 |
| HZ | M | 340326 | 2010 | OBA | 76 | 40 | IIC | 5 | AM | 1 | Press fit | N | 0 | 10 |
